# Circularity in fisheries data weakens real world prediction

**DOI:** 10.1038/s41598-020-63773-3

**Published:** 2020-04-24

**Authors:** Alfredo Giron-Nava, Stephan B. Munch, Andrew F. Johnson, Ethan Deyle, Chase C. James, Erik Saberski, Gerald M. Pao, Octavio Aburto-Oropeza, George Sugihara

**Affiliations:** 1 Scripps Institution of Oceanography, University of California San Diego, 9500 Gilman Dr, CA La Jolla, 92093 USA; 2 National Center for Ecological Analysis and Synthesis, University of California Santa Barbara, 735 State St #300, Santa Barbara, CA 93101 USA; 3 Future Earth, School of Global Environmental Sustainability, Colorado State University, 108 Johnson Dr, Fort Collins, 80523 CO USA; 4 Fisheries Ecology Division. Southwest Fisheries Science Center. National Oceanographic and Atmospheric Administration, 110 Shaffer Rd, Santa Cruz, CA 95060 USA; 5 Department of Ecology and Evolutionary Biology, University of California Santa Cruz, 1156 High Street, Santa Cruz, CA 95064 USA; 6 MarFishEco, Portland, Oregon, USA, London, UK; 7 Salk Institute of Biological Sciences, 10010 North Torrey Pines Rd, La Jolla, CA 92037 USA

**Keywords:** Ecology, Ecological modelling, Population dynamics, Theoretical ecology

## Abstract

The systematic substitution of direct observational data with synthesized data derived from models during the stock assessment process has emerged as a low-cost alternative to direct data collection efforts. What is not widely appreciated, however, is how the use of such synthesized data can overestimate predictive skill when forecasting recruitment is part of the assessment process. Using a global database of stock assessments, we show that Standard Fisheries Models (SFMs) can successfully predict synthesized data based on presumed stock-recruitment relationships, however, they are generally less skillful at predicting observational data that are either raw or minimally filtered (denoised without using explicit stock-recruitment models). Additionally, we find that an equation-free approach that does not presume a specific stock-recruitment relationship is better than SFMs at predicting synthesized data, and moreover it can also predict observational recruitment data very well. Thus, while synthesized datasets are cheaper in the short term, they carry costs that can limit their utility in predicting real world recruitment.

## Introduction

Faced with budget reductions for fisheries science and management worldwide, fisheries programs have experienced pressure to systematically replace or augment observational data programs with less expensive indirect data estimation programs that produce so called synthesized data^1,2^. These programs construct continuous time series from sparse observations of standing stock biomass (SSB) or catch data, using model-based estimates to filter noise from the raw observations and to fill in for times that were not directly sampled^3^. Thus, such estimates are necessarily the product of the model assumptions for how fish populations grow^4^. These synthetic time series are commonly used as a record of stock status and as input data for predicting recruitment (fisheries productivity) which in some cases can directly influence management strategies^5,6^.

A problem arises when data that were synthesized with explicit assumptions about the stock-recruitment relationship (SRR) are then used to make predictions about that relationship as it occurs in the real world. Although models can often be made to fit and predict model-generated data, the accuracy of such models at predicting real world observations is often very low. There is potential for circularity in the overall approach^3,7^, all of which casts doubt about whether a relationship between the SSB and recruitment in nature actually exists^8–10^. Recent studies that find evidence for such a relationship in principle, are beginning to question whether it can be used to improve our practical ability to predict recruitment^11,12^. For example, Pierre *et al*. find positive evidence of a causal relationship between stock size and recruitment, however, they also conclude that recruitment is largely unpredictable using classical models that are based on stock size alone^11^. This finding is supported by Deyle *et al*. who uses a non-parametric nonlinear EDM approach (Empirical DynamicModelling, see Box 1) to find that for Atlantic and Gulf Menhaden recruitment is indeed predictable from year to year, but only when allowing for realistic interdependence of adult stock size with other ecological factors^13^. More recently, Munch *et al*. analyzed a global database with EDM, to find that on average 40% of the variability in recruitment can be explained by previously observed recruitment fluctuations^12^, which according to theory should contain information about the relevant environmental drivers^12,13^.

Here, we examine the Ransom Myers database^14^, a global repository of stock sizes and recruitment estimates for over 600 marine and freshwater fish populations (>100 species) to ask the following questions: (1) How well do standard fisheries models (SFMs) predict the number of recruits when the data are synthesized by an assessment method that incorporates an explicit stock-recruitment model (synthetic data, SD) versus data that come directly from surveys or statistically denoised estimates such as those that come from a sequential population analysis that do not have density dependence built-in (direct data, DD)? And (2) can an equation-free EDM approach provide better predictions than SFMs? We selected all populations from this database with at least 25 years of both stock size and recruitment, representing 134 populations from 36 species, spanning 8 orders. The datasets were classified according to their origin as SD or DD, see *Materials and Methods* for further details.

BOX 1 Empirical dynamic modeling Empirical Dynamic Modeling (EDM) is a data-driven approach to understand mechanisms, identify causal variables and make predictions on complex systems. It relies on extracting system behavior directly from observed time series^15–17^. The rationale for this approach is that dynamical relationships that are too complex or subtle to capture in a simple set of equations can instead be recovered empirically from time series observations^18^. EDM is specifically capable of describing nonlinear, state-dependent interactions (e.g. where the effect of one variable on another can depend on a third or fourth dynamically changing factor etc.). Such interactions are difficult to describe with models having constant coefficients and thus may not yield to standrad model-building approaches (e.g. those that use single-factor experiments to estimate fixed rate parameters)^10^. The essential ideas and concepts of EDM are briefly summarized in three 1-minute videos at http://tinyurl.com/EDM-intro.

**Figure B1:**
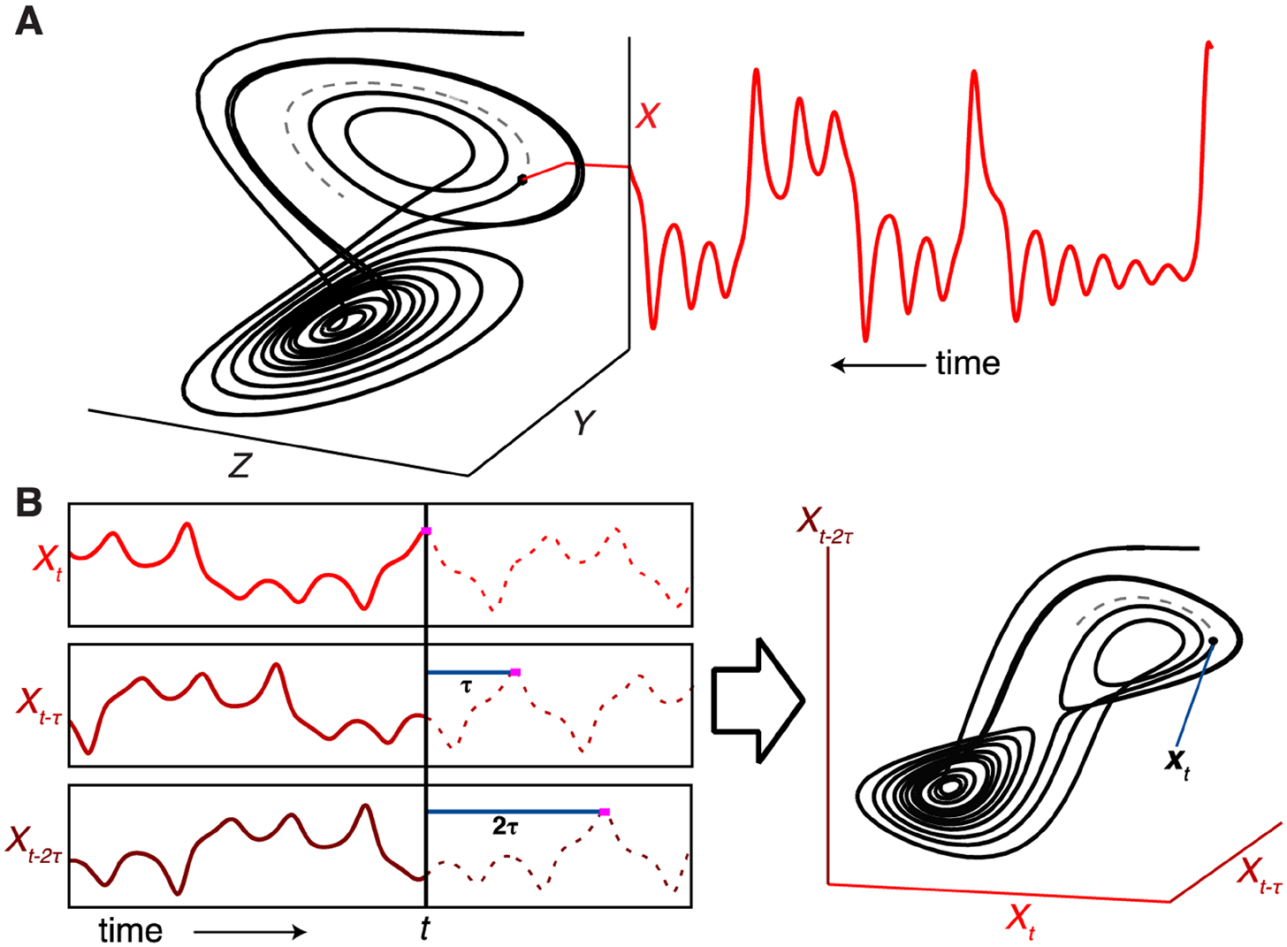
Reconstruction of System Dynamics from a Time Series (using the canonical Lorenz attractor example) (**A**) Projecting the motion of the Lorenz attractor onto the x-axis yields a time series for variable x. (**B**) Successive lags (with time step τ) of the time series x_t_ are plotted as separate coordinates to form a reconstructed (and visually similar) “shadow” attractor that preserves essential mathematical properties of the original system.

The basic premise of EDM is to view a dynamic system from a geometrical perspective – the attractor. The attractor can be generated from some underlying set of equations or from observational time series data, and describes how variables change with respect to each other. Thus, as the system changes over time, its trajectory moves across different points in the state-space, and over time the paths form a geometric attractor (Fig. B1). A time series of a state variable can then be viewed as a projection of the motion on an attractor onto the coordinate axes recording how that variable changes through time. A natural way to view the system is in its native state space where each coordinate axis is an essential active causal variable – this primary form of EDM attractor reconstruction requires multiple time series, one for each active causal variable^16,19,20^. However, Takens theorem^21^ also allows one to construct equivalent attractors using lags of just one time series. Here, time lags of a single variable can serve as proxies for the other variables (Fig. B1).

Again, the primary motivation for using EDM is to address nonlinear, non-equilibrium behavior. In contrast, many statistical methods (e.g. generalized linear models, structural equation modeling, dynamic linear models) rely on describing dynamics in terms of relationships that are constant or fixed in time, rather than in terms of relationships that are interdependent (state dependent) and that vary in time. For example, linear dynamics can occur with a static equilibrium when the dynamics are essentially viewed as stochastic perturbations around a fixed point, as in a typical linear stability analysis. Because nonlinear behavior appears to be ubiquitous in nature, EDM has been applied broadly across domains, from astrophysics^22^, geophysics and climate^23^, to epidemiology^24^, cardiology^25^ and medicine^26,27^.

## Results

**Figure 1:**
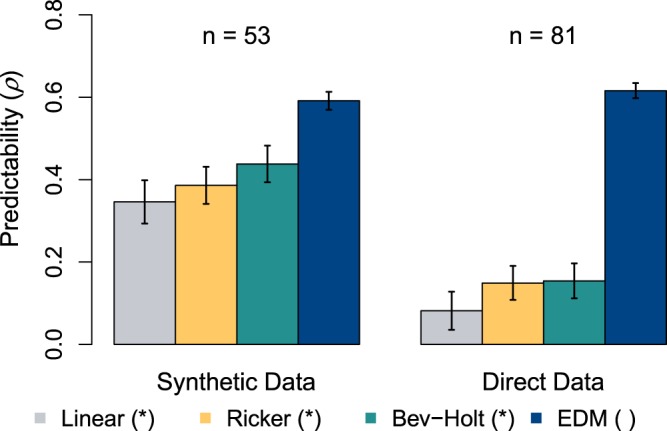
Potential circularity arises when models predict model output (SD). Comparison of predictability of spawner-recruit relationships for three standard fisheries models (Linear, Ricker, and Beverton-Holt) to equation-free EDM (S-maps). The y-axis represents the average predictability as measured by the correlation coefficient between 25 observed data points and their corresponding modeled predictions. The error bars represent the standard error. Asterisk labels next to the assessment method labels indicate a significant difference (P < 0.05) between thepredictability for SD as compared to DD.

SFMs reported higher predictability (higher Pearson correlation coefficient between observed and predicted recruitment, ρ) when using SD as compared to DD. In the case of SD, the average Pearson correlation ρ values were equal to 0.35, 0.38 and 0.43 for the density independent, Ricker and Beverton-Holt models respectively (Fig. 1), with an overall average value of 0.39. In the case of the DD, average ρ values were equal to 0.08, 0.15 and 0.15respectively for the same models (Fig. 1), with an overall average value of 0.13. This represents an overall average decrease of 65% in the predictive capacity of SFMs when using DD versus SD. When confronted with real-world direct data, the reduction in predictability was significant (P < 0.05) for each of the three SFMs.

Regardless of the type of data used, EDM outperformed the three SFMs in predicting the number of recruits. On average, EDM reported Pearson correlation ρ values equal to 0.59 and 0.62 for SD and DD respectively (Fig. 1). We found no significant differences in the predictability of EDM between both types of data (P > 0.05).

## Discussion

The use of synthetic data (SD) can be misleading when SFMs are used to study the stock-recruitment problem. Circularity arises when SD estimated through models is used as input into SFMs to predict SD.

Nonetheless, SD filtered with an explicit Stock-Recruitment model has been a critically useful assessment procedure when applied consistently. The majority of our stock assessment records have been derived this way, which allows us, importantly, to track stock status and productivity, albeit ultimately with a proxy for actual recruitment. Thus the consistent use of this procedure allows for historical comparisons to be made, which has ongoing merit in a management context. However, issues arise in terms of the original objective if we literally want to be able to make forecasts of real-world recruitment to generate management advice. The inability of SFM’s to accomplish this original aim comes in part because the assumptions of the models are uncertain (e.g. the hypothesized functional form of the model, the classic assumption of near-equilibrium linear dynamics, etc. Box 1)^3,9,28,29^. Nonetheless the SFM’s usefulness to provide an internally consistent record on stock status and productivity, though the ability to predict data it has produced should not be taken as validation of the models, and the SD should not be used in place of more direct observations in studies of fisheries recruitment.

As we move forward, if one of our main scientific management objectives is to improve our ability to predict recruitment as a real-world quantity, the potential problems with data circularity need to be recognized and constructively addressed, and alternatives such as EDM need to be considered. EDM derives the dynamic mechanisms and causes directly from the data – a capability that allowed it to accommodate the dynamics introduced to the SD by the SFM’s (Fig. 1). More importantly, EDM was able to perform well with the DD and thus presumably is able to predict recruitment in the real fishery (Fig. 1). EDM methods have previously been used to improve recruitment predictability for a variety of stocks, for example: tuna in the North Pacific^30^, sockeye salmon in the Fraser River system in British Columbia^10^, red snapper in the Gulf of Mexico^31^, and menhaden from the Gulf of Mexico and the Atlantic menhaden^13^. Although these results seem promising, the challenge remains to integrate these benchmark predictions more broadly into specific enacted management schemes, and particularly ones that are sustainably adaptive to non-stationary harvest targets^12^.

## Methods

### Ransom myers database

We compared the predictions of the numbers of recruits through time from stock assessments using Standard Fisheries Models (SFMs)^4^ and an Empirical Dynamic Modelling (EDM) technique known as S-maps^16,19,32^. To do this, we used the Ransom Myers database^14^, a global repository of stock sizes and recruitment estimates for over 600 marine and freshwater fish populations (>100 species). All populations from this database with at least 25 years of both stock size and recruitment data were included in our analysis, representing 134 populations from 36 species, spanning 8 orders. We classified each time series into one of two categories based on the nature of the data: data reanalyzed with an explicit Stock-Recruitment model (synthetic data, SD) (n = 53) and data from direct or statistically denoised observations (direct data, DD) (n = 81). The SD included datasets derived from Biomass Dynamic Models (BDM), while the DD included datasets derived from Sequential Population Analyses (SPA) or Direct Observations (DO). SPA datasets were classified as DD given that they only make an assumption about constant natural mortality to back calculate recruitment from landings data, which does not introduce an explicit assumption for the SR relationship. There were 3 datasets derived from Statistical Catch-at-Age (SCA) analyses that also met the requirement of having at least 25 years of data, however, because no information about whether explicit assumptions about the SR relationship was given, they were not included in our analyses. Supplementary Table I presents a summary of the original method used in each stock assessment, the classification as either SDor DD, and time series’ length.

### Predictability–standard fisheries models (SFMs)

We evaluated the performance of three SFMs to predict the spawner-recruit relationship in the 134 populations from the Ransom Myers database: density independence, Ricker, and Beverton-Holt^4^. These models assume that the number of recruits is a function of the current stock size. All models can be written in the general form ${R}_{t}=\alpha {S}_{t}g({S}_{t})$, where $R$ is recruitment, $S$ is stock size, $\alpha$ is the maximum rate of reproduction, and $g({S}_{t})$ is a function that accounts for density-dependent processes^14^. In the case of the density-independent model, the function $g({S}_{t})=1$ and the model is a straight line that intercepts the origin (0,0) with slope $\alpha$. The Ricker and Beverton-Holt models introduce the term $\beta$, which is proportional to the product of fecundity and density-dependent mortality (see e.g. Quinn & Deriso^33^). The three models are presented below.

1 $${R}_{t}=\alpha {S}_{t}\,\mbox{--}\,{\rm{Density}}\,{\rm{independent}}$$

2 $${R}_{t}=\alpha {S}_{t}{e}^{-\beta {S}_{t}}\,\mbox{--}\,{\rm{Ricker}}$$

3 $${R}_{t}=\alpha {S}_{t}\left(\frac{1}{1+\beta {S}_{t}}\right)\,\mbox{--}\,{\rm{Beverton}}-{\rm{Holt}}$$

The Ricker and Beverton-Holt models were fitted on a log scale, re-written so that ${y}_{t}=\,\mathrm{ln}\,[{R}_{t}/{S}_{t}]$^33^. All models were fit using the function ‘fminsearch’ in Matlab R2015b.

To calculate the predictability achieved by each model, we performed leave-one-out cross validation.The minimum length of any time series in our dataset was 25 years; thus, to ensure sample sizes were consistent across models, for time series with more than 25 points, we randomly selected 25 points as targets to be predicted. For each prediction on a target, the point one timestep before the target and 23 other, randomly selected points were used to fit model parameters, and used to make a prediction on the target. For time series with exactly 25 points, all points were used as targets to be predicted, and the other 24 points were used to fit model parameters on each iteration. Even though the Ricker and Beverton-Holt models were fitted on a log scale, all predictions were made in the original recruitment scale. We then calculated the predictability (ρ) as the Pearson correlation coefficient between the 25 observations and their respective predicted values.

### Predictability–empirical dynamic modelling (EDM)

EDM is based on the idea that time series are one-dimensional projections (a time record of some coordinate or variable) of a dynamic system (see introductory video https://youtu.be/fevurdpiRYg). If there are “n” relevant variables the trajectory produced as the system evolves in this n-dimensional space would produce a geometric shape or an “attractor”. Following the trajectories at locations on an attractor nearby to a current state allows one to predict future states^15,16^. Because in practice we may not know what all the relevant variables are or even how many relevant variables there are (the “n” of the n-dimensional coordinate space), we can use Takens’ Theorem^21^ to construct a shadow version of the original attractor from a single time series or single variable that we want to predict. Thus, assuming that the single time series is ${x}_{t}$, one can reconstruct a “shadow” version of the original attractor by using lagged time series (eg. ${x}_{t-1}$, ${x}_{t-2}$) as proxies for other unknown time series of the same system and predict future values of ${x}_{t}$. Again, the number of time-lagged proxies required (the embedding dimension) corresponds to the number of active causal variables – or number of coordinates required to embed the attractor. The principles and mechanics of Takens’ theorem and EDM are illustrated in Box 1 and further explained in Deyle and Sugihara^34^ and Sugihara *et al*.^17^ and in a series of short animations (http://tinyurl.com/EDM-intro).

Although it is possible to construct an attractor from a single time series (univariate reconstruction), predictability can often be improved by using multiple time series of different active causal variables measured from the same system (multivariate reconstruction)^16,35^. For example, for modelling fish stocks a useful multivariate reconstruction may involve a time series for fish stock biomass (${S}_{t}$), another time series for the number of recruits (${R}_{t}$), as well as time-lagged time series of both ${S}_{t}$ and ${R}_{t}$. We tested all the possible combinations of these 6 time series (${R}_{t}$, ${R}_{t-1},\,{R}_{t-2},\,{S}_{t},{S}_{t-1},\,{S}_{t-2}$) to make multivariate reconstructions, going from using 1 to 6 time series at a time.

This generalized embedding scheme is used to make forecasts using S-maps^32^, which is a standard weighted kernel regression scheme that controls local weights with a tuning parameter $\theta$. When $\theta$ = 0 all the points on the attractor are equally weighted to generate a single global linear map. When $\theta$ > 0 more weight is given to points nearby each predictee on the attractor, so that the map produced for each forecast differs with location on the attractor (map varies with the system state). Finding that prediction improves for any $\theta$ > 0 indicates curvature (nonlinearity) in the attractor. All our results report the predictability (ρ) achieved when $\theta$ is optimized.

Thus, as with the SFMs, to avoid overfitting we perform a leave-one-out cross validation by excluding the single time point that we are trying to predict from the data used to build the forecast model. We calculate ρ as the maximum Pearson correlation coefficient between the observations and their respective predicted values for each of the 134 analyzed fish stocks. All analyses were performed using the *rEDM *package in CRAN (*v. 0.7.2*).

### Differences in predictability between SD and DD

An unpaired t-test was used to test whether a particular model’s predictions were significantly different when using SD versus DD. In Fig. 1 an asterisk indicates when the differences in predictability (ρ) between SD versus DD for each model type are significant (P < 0.05).

## Supplementary information

Supplementary Information.

## Data Availability

Should the manuscript be accepted, the data supporting the results will be archived in an appropriate public repository such as Dryad or Figshare and the data DOI will be included at the end of the article.
