# Supplementary material for: Circularity in fisheries data weakens real world prediction
Source: Sci Rep. 2020 Apr 24;10:6977. doi: 10.1038/s41598-020-63773-3 (PMC7181812; doi:10.1038/s41598-020-63773-3)
Supplement: Supplementary file 1 — Supplementary Information. [file 41598_2020_63773_MOESM1_ESM.docx]

**Supplementary materials**

*Supplementary Table I.*

List of stocks from the RAM Legacy database with 25 or more years of data. The table shows the scientific name of the species, original collection method (Survey – direct observations, SPA – Sequential Population Analysis, BDM – Biomass Dynamics Model), method category given in this study – Data reanalyzed with an explicit Stock-Recruitment model (synthetic data, SD) and data from direct or statistically denoised observations (direct data, DD) – length of the time series in number of years, and the predictability (ρ) achieved by each of the 3 SFMs and EDM.

| **ID** | **Species** | **Method** | **Method category** | **Length (yr)** | **Linear** | **Ricker** | **Beverton Holt** | **EDM** |
| --- | --- | --- | --- | --- | --- | --- | --- | --- |
| 1 | Alosa pseudoharengus | Survey | DD | 45 | 0.41 | 0.35 | 0.08 | 0.87 |
| 3 | Alosa pseudoharengus | Survey | DD | 35 | 0.50 | 0.67 | 0.67 | 0.79 |
| 11 | Engraulis encrasicolus | SPA | DD | 27 | 0.81 | 0.79 | 0.82 | 0.78 |
| 13 | Salmo salar | Survey | DD | 37 | -0.43 | -0.05 | -0.21 | 0.68 |
| 14 | Salmo salar | Survey | DD | 38 | 0.38 | 0.25 | 0.35 | 0.51 |
| 19 | Harpodon nehereus | Survey | DD | 29 | 0.95 | 0.96 | 0.97 | 0.80 |
| 24 | Thunnus obesus | SPA | DD | 35 | 0.22 | 0.23 | -0.33 | 0.74 |
| 25 | Thunnus obesus | SPA | DD | 26 | -0.56 | -0.35 | 0.01 | 0.47 |
| 26 | Thunnus obesus | SPA | DD | 25 | 0.13 | 0.10 | -0.40 | 0.74 |
| 34 | Oncorhynchus tshawytscha | BDM | SD | 26 | -0.09 | -0.15 | -0.21 | 0.89 |
| 40 | Oncorhynchus keta | BDM | SD | 38 | -0.50 | -0.04 | -0.44 | 0.31 |
| 41 | Oncorhynchus keta | BDM | SD | 30 | 0.25 | 0.27 | 0.34 | 0.75 |
| 43 | Oncorhynchus keta | BDM | SD | 28 | 0.45 | 0.43 | 0.48 | 0.45 |
| 44 | Oncorhynchus keta | BDM | SD | 30 | -0.33 | -0.12 | 0.06 | 0.39 |
| 45 | Oncorhynchus keta | BDM | SD | 25 | -0.38 | 0.12 | 0.10 | 0.73 |
| 46 | Oncorhynchus keta | BDM | SD | 25 | -0.07 | 0.02 | 0.18 | 0.46 |
| 47 | Scomber japonicus | SPA | DD | 36 | 0.49 | 0.35 | 0.41 | 0.40 |
| 48 | Gadus morhua | SPA | DD | 35 | 0.70 | 0.53 | 0.60 | 0.62 |
| 49 | Gadus morhua | SPA | DD | 28 | 0.75 | 0.70 | 0.73 | 0.89 |
| 50 | Gadus morhua | SPA | DD | 27 | 0.29 | 0.19 | 0.39 | 0.77 |
| 53 | Gadus morhua | SPA | DD | 31 | -0.04 | 0.16 | 0.20 | 0.75 |
| 54 | Gadus morhua | SPA | DD | 41 | -0.27 | -0.42 | 0.08 | 0.59 |
| 55 | Gadus morhua | SPA | DD | 33 | -0.21 | -0.32 | 0.15 | 0.83 |
| 56 | Gadus morhua | SPA | DD | 45 | 0.11 | 0.17 | -0.19 | 0.42 |
| 62 | Gadus morhua | SPA | DD | 33 | -0.38 | -0.23 | -0.31 | 0.54 |
| 63 | Gadus morhua | Survey | DD | 35 | 0.47 | 0.48 | 0.58 | 0.65 |
| 64 | Gadus morhua | SPA | DD | 68 | -0.10 | 0.07 | -0.08 | 0.41 |
| 65 | Gadus morhua | SPA | DD | 27 | -0.62 | -0.15 | -0.28 | 0.54 |
| 67 | Gadus morhua | SPA | DD | 43 | 0.24 | 0.37 | 0.33 | 0.59 |
| 68 | Gadus morhua | SPA | DD | 45 | -0.04 | 0.26 | 0.09 | 0.66 |
| 69 | Gadus morhua | SPA | DD | 59 | -0.28 | -0.01 | -0.10 | 0.47 |
| 70 | Gadus morhua | SPA | DD | 30 | 0.16 | 0.25 | 0.26 | 0.43 |
| 73 | Gadus morhua | SPA | DD | 27 | 0.12 | 0.05 | -0.65 | 0.41 |
| 77 | Promoxis annularis and nigromaculatus | Survey | DD | 28 | -0.22 | -0.22 | -0.55 | 0.52 |
| 82 | Coilia dussumieri | SPA | DD | 26 | 0.84 | 0.78 | 0.68 | 0.61 |
| 84 | Melanogrammus aeglefinus | SPA | DD | 38 | 0.56 | 0.47 | 0.70 | 0.67 |
| 86 | Melanogrammus aeglefinus | SPA | DD | 26 | -0.83 | -0.60 | -0.05 | 0.76 |
| 87 | Melanogrammus aeglefinus | SPA | DD | 68 | 0.37 | 0.33 | 0.53 | 0.76 |
| 88 | Melanogrammus aeglefinus | SPA | DD | 33 | 0.04 | 0.26 | -0.06 | 0.64 |
| 89 | Melanogrammus aeglefinus | SPA | DD | 28 | -0.35 | -0.27 | -0.03 | 0.33 |
| 90 | Melanogrammus aeglefinus | SPA | DD | 44 | 0.10 | 0.26 | 0.28 | 0.51 |
| 91 | Melanogrammus aeglefinus | SPA | DD | 32 | -0.12 | -0.40 | -0.21 | 0.88 |
| 92 | Melanogrammus aeglefinus | SPA | DD | 66 | -0.32 | -0.40 | -0.40 | 0.58 |
| 94 | Melanogrammus aeglefinus | SPA | DD | 29 | -0.39 | -0.34 | -0.58 | 0.65 |
| 108 | Clupea harengus | SPA | DD | 38 | -0.13 | 0.32 | -0.06 | 0.58 |
| 110 | Clupea harengus | SPA | DD | 65 | 0.66 | 0.51 | 0.44 | 0.75 |
| 116 | Clupea harengus | Survey | DD | 48 | 0.15 | 0.24 | 0.22 | 0.45 |
| 122 | Clupea harengus | SPA | DD | 49 | 0.29 | 0.37 | 0.29 | 0.58 |
| 124 | Clupea harengus | SPA | DD | 44 | 0.14 | 0.22 | 0.18 | 0.66 |
| 125 | Clupea harengus | SPA | DD | 41 | 0.24 | 0.53 | 0.44 | 0.56 |
| 126 | Clupea harengus | SPA | DD | 38 | -0.32 | 0.16 | -0.05 | 0.48 |
| 127 | Clupea harengus | SPA | DD | 38 | -0.30 | -0.29 | -0.07 | 0.25 |
| 128 | Clupea harengus | SPA | DD | 38 | 0.05 | -0.10 | 0.31 | 0.60 |
| 129 | Clupea harengus | SPA | DD | 38 | -0.45 | -0.13 | -0.32 | 0.74 |
| 135 | Clupea harengus | SPA | DD | 38 | -0.04 | 0.07 | 0.24 | 0.64 |
| 136 | Clupea harengus | SPA | DD | 38 | -0.35 | -0.34 | -0.46 | 0.61 |
| 140 | Penaeus orientalis | SPA | DD | 26 | -0.03 | 0.11 | 0.11 | 0.16 |
| 142 | Salvelinus namaycush | SPA | DD | 40 | 0.78 | 0.80 | 0.76 | 0.91 |
| 143 | Scomber scombrus | Survey | DD | 36 | 0.54 | 0.64 | 0.61 | 0.46 |
| 144 | Trachurus mediterraneus | SPA | DD | 45 | -0.16 | -0.16 | -0.04 | 0.67 |
| 148 | Brevoortia tyrannus | SPA | DD | 40 | 0.05 | 0.37 | 0.20 | 0.71 |
| 149 | Brevoortia patronus | SPA | DD | 26 | 0.36 | 0.38 | 0.40 | 0.67 |
| 153 | Sebastes alutus | SPA | DD | 30 | -0.62 | -0.53 | 0.03 | 0.56 |
| 154 | Sebastes alutus | SPA | DD | 31 | 0.23 | 0.07 | 0.40 | 0.66 |
| 155 | Sebastes alutus | SPA | DD | 26 | -0.81 | -0.62 | -0.03 | 0.79 |
| 156 | Sebastes alutus | SPA | DD | 29 | 0.43 | 0.46 | 0.52 | 0.42 |
| 158 | Esox lucius | SPA | DD | 35 | 0.15 | 0.22 | 0.16 | 0.33 |
| 159 | Esox lucius | SPA | DD | 35 | -0.09 | 0.01 | -0.03 | 0.43 |
| 160 | Oncorhynchus gorbuscha | BDM | SD | 25 | 0.60 | 0.63 | 0.63 | 0.51 |
| 189 | Oncorhynchus gorbuscha | BDM | SD | 25 | 0.79 | 0.80 | 0.81 | 0.72 |
| 193 | Oncorhynchus gorbuscha | BDM | SD | 27 | 0.10 | -0.18 | 0.33 | 0.38 |
| 194 | Oncorhynchus gorbuscha | BDM | SD | 25 | 0.15 | 0.15 | 0.00 | 0.28 |
| 197 | Oncorhynchus gorbuscha | BDM | SD | 42 | 0.10 | 0.26 | 0.25 | 0.65 |
| 200 | Oncorhynchus gorbuscha | BDM | SD | 34 | 0.48 | 0.54 | 0.60 | 0.57 |
| 202 | Oncorhynchus gorbuscha | BDM | SD | 27 | 0.52 | 0.55 | 0.60 | 0.72 |
| 203 | Oncorhynchus gorbuscha | BDM | SD | 27 | 0.25 | 0.24 | 0.33 | 0.67 |
| 207 | Oncorhynchus gorbuscha | BDM | SD | 27 | -0.66 | -0.11 | -0.05 | 0.30 |
| 208 | Oncorhynchus gorbuscha | BDM | SD | 34 | 0.05 | -0.01 | 0.31 | 0.57 |
| 211 | Oncorhynchus gorbuscha | BDM | SD | 27 | 0.04 | 0.07 | 0.47 | 0.55 |
| 212 | Oncorhynchus gorbuscha | BDM | SD | 27 | 0.48 | 0.51 | 0.56 | 0.85 |
| 217 | Pleuronectes platessa | SPA | DD | 28 | -0.17 | -0.39 | -0.22 | 0.62 |
| 219 | Pleuronectes platessa | SPA | DD | 35 | 0.03 | 0.08 | -0.37 | 0.48 |
| 221 | Pollachius virens | SPA | DD | 32 | -0.26 | -0.12 | -0.07 | 0.52 |
| 222 | Pollachius virens | SPA | DD | 32 | 0.20 | 0.20 | -0.38 | 0.55 |
| 223 | Pollachius virens | SPA | DD | 32 | 0.12 | 0.14 | 0.35 | 0.50 |
| 224 | Pollachius virens | SPA | DD | 33 | 0.21 | 0.27 | 0.55 | 0.55 |
| 225 | Pollachius virens | SPA | DD | 30 | -0.01 | 0.27 | 0.24 | 0.73 |
| 233 | Anoplopoma fimbria | SPA | DD | 25 | 0.01 | 0.07 | 0.19 | 0.54 |
| 236 | Sardinops sagax | SPA | DD | 31 | 0.05 | 0.13 | 0.13 | 0.80 |
| 239 | Sardinops sagax | SPA | DD | 31 | 0.70 | 0.73 | 0.71 | 0.78 |
| 245 | Thunnus maccoyii | SPA | DD | 45 | 0.75 | 0.77 | 0.77 | 0.91 |
| 249 | Merluccius bilinearis | SPA | DD | 33 | 0.83 | 0.82 | 0.85 | 0.95 |
| 250 | Merluccius bilinearis | SPA | DD | 33 | 0.53 | 0.59 | 0.57 | 0.92 |
| 251 | Oncorhynchus nerka | BDM | SD | 39 | 0.86 | 0.86 | 0.88 | 0.77 |
| 252 | Oncorhynchus nerka | BDM | SD | 43 | 0.59 | 0.68 | 0.66 | 0.82 |
| 253 | Oncorhynchus nerka | BDM | SD | 39 | 0.35 | 0.35 | 0.42 | 0.57 |
| 254 | Oncorhynchus nerka | BDM | SD | 43 | 0.43 | 0.63 | 0.55 | 0.55 |
| 255 | Oncorhynchus nerka | BDM | SD | 38 | 0.27 | 0.28 | 0.25 | 0.61 |
| 256 | Oncorhynchus nerka | BDM | SD | 33 | 0.39 | -0.28 | 0.46 | 0.56 |
| 257 | Oncorhynchus nerka | BDM | SD | 45 | 0.75 | 0.80 | 0.80 | 0.66 |
| 258 | Oncorhynchus nerka | BDM | SD | 38 | -0.34 | 0.14 | -0.19 | 0.75 |
| 259 | Oncorhynchus nerka | BDM | SD | 39 | 0.63 | 0.68 | 0.72 | 0.54 |
| 260 | Oncorhynchus nerka | BDM | SD | 43 | 0.69 | 0.76 | 0.70 | 0.61 |
| 262 | Oncorhynchus nerka | BDM | SD | 32 | 0.55 | 0.53 | 0.69 | 0.35 |
| 264 | Oncorhynchus nerka | BDM | SD | 39 | 0.61 | 0.60 | 0.62 | 0.76 |
| 267 | Oncorhynchus nerka | BDM | SD | 39 | 0.77 | 0.74 | 0.90 | 0.97 |
| 268 | Oncorhynchus nerka | BDM | SD | 43 | 0.94 | 0.91 | 0.91 | 0.85 |
| 270 | Oncorhynchus nerka | BDM | SD | 39 | -0.01 | 0.21 | -0.05 | 0.63 |
| 271 | Oncorhynchus nerka | BDM | SD | 62 | 0.35 | 0.38 | 0.28 | 0.45 |
| 274 | Oncorhynchus nerka | BDM | SD | 38 | 0.67 | 0.67 | 0.69 | 0.66 |
| 275 | Oncorhynchus nerka | BDM | SD | 38 | 0.69 | 0.80 | 0.76 | 0.56 |
| 276 | Oncorhynchus nerka | BDM | SD | 42 | 0.81 | 0.85 | 0.78 | 0.51 |
| 277 | Oncorhynchus nerka | BDM | SD | 32 | 0.65 | 0.49 | 0.67 | 0.54 |
| 279 | Oncorhynchus nerka | BDM | SD | 38 | 0.70 | 0.58 | 0.74 | 0.74 |
| 280 | Oncorhynchus nerka | Survey | DD | 32 | 0.17 | 0.23 | 0.28 | 0.63 |
| 283 | Oncorhynchus nerka | BDM | SD | 42 | -0.07 | -0.03 | 0.01 | 0.55 |
| 284 | Oncorhynchus nerka | BDM | SD | 39 | 0.23 | 0.31 | 0.24 | 0.66 |
| 285 | Oncorhynchus nerka | BDM | SD | 45 | 0.37 | 0.40 | 0.43 | 0.26 |
| 287 | Oncorhynchus nerka | BDM | SD | 39 | 0.63 | 0.58 | 0.53 | 0.62 |
| 288 | Oncorhynchus nerka | BDM | SD | 43 | 0.33 | 0.66 | 0.42 | 0.61 |
| 289 | Oncorhynchus nerka | BDM | SD | 39 | 0.72 | 0.71 | 0.83 | 0.60 |
| 290 | Oncorhynchus nerka | BDM | SD | 43 | 0.78 | 0.80 | 0.83 | 0.46 |
| 293 | Oncorhynchus nerka | BDM | SD | 33 | -0.12 | 0.12 | 0.14 | 0.41 |
| 294 | Oncorhynchus nerka | BDM | SD | 30 | 0.19 | -0.12 | 0.28 | 0.64 |
| 296 | Oncorhynchus nerka | BDM | SD | 38 | 0.59 | 0.63 | 0.59 | 0.52 |
| 298 | Oncorhynchus nerka | BDM | SD | 39 | 0.78 | 0.04 | 0.82 | 0.67 |
| 299 | Oncorhynchus nerka | BDM | SD | 43 | 0.43 | 0.50 | 0.44 | 0.66 |
| 301 | Oncorhynchus nerka | BDM | SD | 40 | -0.08 | 0.18 | 0.05 | 0.50 |
| 305 | Solea vulgaris | SPA | DD | 36 | -0.27 | -0.19 | -0.29 | 0.59 |
| 311 | Sprattus sprattus | SPA | DD | 43 | 0.55 | 0.55 | 0.54 | 0.64 |
| 323 | Merlangius merlangus | SPA | DD | 27 | -0.34 | -0.30 | -0.18 | 0.49 |
| 324 | Merlangius merlangus | SPA | DD | 74 | -0.37 | -0.35 | -0.29 | 0.28 |
| 326 | Merlangius merlangus | SPA | DD | 27 | -0.69 | -0.04 | -0.05 | 0.73 |
| 331 | Thunnus albacares | SPA | DD | 25 | 0.28 | 0.05 | 0.39 | 0.70 |
